# Applications of Artificial Intelligence Methods for the Prediction of Osteoporotic Fractures

**DOI:** 10.3390/life13081738

**Published:** 2023-08-13

**Authors:** Dorota Lis-Studniarska, Marta Lipnicka, Marcin Studniarski, Robert Irzmański

**Affiliations:** 1 Central Clinical Hospital, Medical University of Łódź, Pomorska 251, 92-213 Łódź, Poland; 2 Faculty of Mathematics and Computer Science, University of Łódź, Banacha 22, 90-238 Łódź, Poland; marta.lipnicka@wmii.uni.lodz.pl (M.L.); marcin.studniarski@wmii.uni.lodz.pl (M.S.); 3 Department of Internal Medicine, Rehabilitation and Physical Medicine, Medical University of Łódź, plac Gen. Józefa Hallera 1, 90-645 Łódź, Poland; robert.irzmanski@umed.lodz.pl

**Keywords:** osteoporosis, fractures, risk factors, medical records, artificial neural networks, logistic regression

## Abstract

*Background*: Osteoporosis is a socio-economic problem of modern aging societies. Bone fractures and the related treatments generate the highest costs. The occurrence of osteoporotic fractures is a cause of chronic disability, many complications, reduced quality of life, and often premature death. *Aim of the study:* The aim of the study was to determine which of the patient’s potential risk factors pertaining to various diseases and lifestyle have an essential impact on the occurrence of low-energy fractures and the hierarchy of these factors. *Methods:* The study was retrospective. The documentation of 222 patients (206 women and 16 men) from an osteoporosis treatment clinic in Łódź, Poland was analyzed. Each patient was described by a vector consisting of 27 features, where each feature was a different risk factor. Using artificial neural networks, an attempt was made to create a model that, based on the available data, would be able to predict whether the patient would be exposed to low-energy fractures. We developed a neural network model that achieved the best result for the testing data. In addition, we used other methods to solve the classification problem, i.e., correctly dividing patients into two groups: those with fractures and those without fractures. These methods were logistic regression, *k*-nearest neighbors and SVM. *Results:* The obtained results gave us the opportunity to assess the effectiveness of various methods and the importance of the features describing patients. Using logistic regression and the recursive elimination of features, a ranking of risk factors was obtained in which the most important were age, chronic kidney disease, neck T-score, and serum phosphate level. Then, we repeated the learning procedure of the neural network considering only these four most important features. The average mean squared error on the test set was about 27% for the best variant of the model. *Conclusions:* The comparison of the rankings with different numbers of patients shows that the applied method is very sensitive to changes in the considered data (adding new patients significantly changes the result). Further cohort studies with more patients and more advanced methods of machine learning may be needed to identify other significant risk factors and to develop a reliable fracture risk system. The obtained results may contribute to the improved identification patients at risk of low-energy fractures and early implementation of comprehensive treatment.

## 1. Introduction

Osteoporosis is a common disease that causes bones to become abnormally weakened and easily broken. As the authors of [1] noted, fractures are the most important complication of osteoporosis. Therefore, estimating the risk of fractures in different populations is an essential topic of research. Osteoporotic fractures are those of the proximal femur, proximal humerus, distal radius, and vertebrae. Women after menopause are at a high risk for osteoporosis due to lower levels of estrogen, because this hormone helps to maintain bone mass. Men suffer from osteoporosis less often than women, but they are at greater risk of dying in the first year after hip fracture.

Osteoporosis is called the “silent bone thief”. It goes unnoticed, and often its first symptom may be a bone fracture as a result of a fall from one’s own height or without any injury, a so-called low-energy fracture.

The diagnosis for a particular patient is based on densitometry and the occurrence of fractures. In Table 1, we present the classification for osteoporosis in postmenopausal women and men over 50 according to the World Health Organization [2].

The Fracture Risk Assessment Tool (FRAX) [3] is a commonly used method for calculation of the 10-year probability of an osteoporotic fracture; however, it has some shortcomings. In particular, when entering data into the calculator, it is not possible to grade them. Moreover, FRAX underestimates fracture risk for patients with certain diseases [4]. In [5], it was shown that a high risk of fracture according to FRAX was usually associated with a densitometric diagnosis of osteoporosis. It was also observed that FRAX risk scores underestimated actual hip fracture incidence in the lowest deciles of scores and overestimated observed hip fracture incidence in the highest deciles of scores [6].

The following risk factors are taken into account in FRAX: age, sex, weight, height, previous fracture, parent fractured hip, current smoking, glucocorticoids, rheumatoid arthritis, secondary osteoporosis, alcohol use of three or more units per day, and femoral neck bone mineral density (BMD). In case of the absence of BMD, the body mass index (BMI) can be used. There are other risk factors which are not considered in FRAX but are discussed in the literature, for example, in [7]. Therefore, it is important to investigate the influence of these factors on the probability of fractures.

Most osteoporotic fractures—almost 60%—may be asymptomatic and may go undiagnosed. Radiological examination is performed in the lateral projection in the Th4–L4 range. With the aid of X-ray morphometry, the heights of the vertebra in the posterior, middle, and anterior parts are measured. A 20% reduction in the vertebra height is used as the fracture threshold. In disputed cases, a CT scan should be performed; however, this exposes the patient to high doses of X-rays. VFA (vertebral fracture assessment) spinal morphometry using fan beam devices is another application of DXA for assessment of the Th6–L4 vertebrae. This test can be used to evaluate moderate to severe fractures. However, it shows only 50% sensitivity for first-degree fractures.

Currently, solutions are being sought that would help identify risk factors for fractures in patients eligible for interventions to prevent or reduce the risk of osteoporotic fractures. The occurrence of a of low-energetic fracture of the proximal femur or vertebrae increases the risk of another, especially within 2 years. These patients especially should receive multidisciplinary care. The need for treatment, including pharmacotherapy and rehabilitation, is emphasized, especially in patients with fractures and with a high (≥10%) fracture risk over 10 years, calculated using the FRAX calculator.

The motivation for this study was the need to test which of the patient’s potential risk factors pertaining to various diseases and lifestyle, including those not included in FRAX or FRAXplus [8], have an essential impact on the development of osteoporosis and occurrence of low-energy fractures and the hierarchy of these factors. In the Polish population, the percentage of patients receiving an adequate pharmacological treatment had been very small, i.e., about 6% according to the National Health Fund report from 2019 [2]. Low-energy fractures cause suffering, handicap, and the increase costs of medical care.

Another purpose of this study was to examine the possibility of applying statistical methods and basic artificial neural networks (ANNs) to predict osteoporotic fractures. This research involved the use of various artificial intelligence (AI) methods, including ANNs, to analyze the medical data of 222 patients from an osteoporosis treatment clinic in Łódź, Poland. An attempt was made to create a model that, based on the available data, would be able to predict whether the patient would be exposed to the occurrence of low-energy fractures. If such a model can be created, it can be used as a helpful element in medical diagnostics and treatment.

First, a pattern for the ANN was defined, being a collection of information for one specific patient regarding 27 possible risk factors, and the history of fractures. The next step was to build a network model and execute the learning procedure. The last step was to check the effectiveness of the network. This was performed on a set of data other than the data used in the learning process.

## 2. Related Work

In many published papers, the authors discuss the use of different methods of artificial intelligence that help diagnose osteoporosis and determine the risk of fractures. First, we mention two review papers [7,9]. The authors of [7] collected 50 publications devoted to using AI for the identification of risk groups for osteoporosis and fractures. In [7] (Table 8), they listed 48 risk factors for fractures and 12 AI methods used by different researchers. The review [9] quoted 58 papers, of which 15 (published in 2017–2019) were discussed in more detail, describing the basic characteristics of the conducted studies, the AI methods used, what the authors wanted to predict, and the results obtained. In the works reviewed, the input data were mainly the results of densitometry or X-ray examinations, and there were no other risk factors taken into account, e.g., related to the lifestyle of a patient.

In [6], the authors compared three assessment tools (FRAX, Garvan, and QFracture) and two simple classifiers (one based on femoral neck BMD T-score only and another based on age plus femoral neck BMD T-score), evaluating their performance in predicting osteoporotic fractures in older men. They concluded that the age plus femoral neck BMD T-score classifier was as good as more complex assessment tools.

In [10], neural networks and deep learning were applied to classify X-ray bone images in order to detect osteoporosis. The authors of [11] studied the influence of age, sex, height, and weight of a patient on the risk of fractures. They tested 20 different classifiers, which made it possible to select the most important factors which would enable them to classify patients into one of two groups (osteoporosis and no osteoporosis). The same four factors were considered in [12,13], where artificial neural networks were applied to predict fractures. In [14], a random forest algorithm was applied to evaluate the impact of 15 factors on fracture risk. These factors included, for example, vitamin D level, smoking, and excessive coffee drinking.

The authors of [15] used data mining methods to acquire knowledge from medical records in order to predict the risk of osteoporosis for a patient without using the BMD measurement. They considered six uncontrollable risk factors and seven controllable risk factors. The controllable factors were associated with diet and lifestyle. The aim of paper [16] was to construct a hybrid classifier model that discriminated an osteoporotic patient from a healthy one based on BMD values. In [17], fuzzy sets were applied to build an expert system to divide patients into three groups: osteopenia, osteoporosis, and normal condition. In [18], fuzzy sets were used in connection with neural networks to diagnose osteoporosis, where the authors considered 19 possible risk factors. The authors of [19] used fuzzy sets, a genetic algorithm, and a decision tree algorithm to detect osteoporosis; here, the following risk factors were taken into account: age, age of menopause, coffee consumption, BMD, and BMI.

In [20,21,22], the authors applied several different AI methods to estimate the risk of osteoporosis for different populations of patients (for example, in [21], for older Vietnamese women). In [20], six AI models were compared with assessment tools such as FRAX and OSTA (Osteoporosis Self-Assessment Tool for Asians), whereas in [21], four AI models were compared with OSTA. In [22], eight classification algorithms were used to divide people into three groups: healthy, osteopenia, and osteoporosis. The results were compared with the diagnosis based on BMD measurements. One can conclude from the analysis in these three papers that different AI methods have the best performance for specific groups of patients, and there is no single method that is best suited for all.

In [23], the authors compared FRAX and the following machine learning models for estimating the risk of fractures: CatBoost, SVM, and logistic regression. The only algorithm that had better performance than FRAX was CatBoost.

A recent paper [24] describes a new deep-learning model for prediction of osteoporosis, which is interpretable in the sense that it provides a risk assessment for each patient individually with an explanation of the influence of the corresponding risk factors.

## 3. Materials and Methods

### 3.1. Methods of Data Collection

The documentation of 222 patients of the Osteoporosis Treatment Clinic (OTC) at the Central Clinical Hospital in Łódź, Poland was analyzed. The patients were aged 28 to 95, female and male Caucasian race, residing in the territory of the Republic of Poland and registered in the Clinic. From the patient cards, the following data were copied into an Excel table: fractures, chronic diseases, surgeries, medications, lifestyle (diet, smoking, alcohol consumption, physical activity, and sun exposure), age of last menstruation (in post-menopausal women), and history of fractures of the proximal end of the femur in the immediate family.

Due to the T-score result in the densitometry test and the presence of osteoporotic fractures, patients were assigned to one of four groups: 1—osteoporosis with pathological fractures, ICD-10 code: M80; 2—osteoporosis without pathological fractures: M81; 3—osteopenia (other disorders of bone mineralization and structure): M85; 4—other persons not qualified for the above-mentioned three groups.

The results of the densitometric examination of the L1–L4 vertebrae and the proximal femoral epiphysis, as well as laboratory tests of the level of 25 OH vitamin D3, calcium, and phosphates in the blood, were taken from the electronic documentation. The above-mentioned tests were performed in a hospital facility. These data were also entered into the table.

The study was conducted according to the guidelines of the Declaration of Helsinki. The Bioethics Committee at the Medical University of Łódź has approved this research. Patients’ informed consent was waived due to the retrospective nature of the study.

### 3.2. Description of Risk Factors

Below, we describe the risk factors that were taken into account. They were chosen on the basis of their appearance in the literature or because they were regarded as important by the OTC doctors. We provide brief explanations of why we consider these factors important.

**Sex**. It is known that women suffer from osteoporosis more often than men. After menopause, women experience significant bone loss: 1–2% per year. Estrogen deficiency causes an increase in bone remodeling, which makes the bones weaker. It is estimated that the risk of hip fractures for women aged over 70 is 16–18%. Men have a higher peak bone mass at the end of the third decade of life. Therefore, BMD values indicating osteoporosis and the risk of fractures associated with bone loss will be reached later. Although the risk of hip fracture for men over 70 is about 5–6%, the mortality rate after osteoporotic fractures is twice as high as for women. However, osteoporosis in men is often underestimated by physicians [25].

**Age**. A person reaches peak bone mass in the third decade of life. Then, after a period of stabilization, BMD slowly decreases (after the age of 50, 0.5–1% per year). Therefore, the risk of fractures due to osteoporosis increases with age. It is estimated that osteoporosis affects approximately 1/10 of women aged 60, 1/5 of women aged 70, 2/5 of women aged 80, and 2/3 of women aged 90 [26].

**Body mass index (BMI)**. BMI is calculated from the formula: BMI = (weight)/(height)${}^{2}$. BMI can be used in the FRAX calculator if BMD is not known. The estimated probability of fracture increases as BMI decreases [3].

**Last menstrual period** (for women only, age in years). The research reported in [27] showed a high risk for osteoporotic fractures in non-obese women who have untreated premature menopause.

**Alcohol**. Alcohol can increase the excretion of calcium from the body. Excessive alcohol consumption may have a toxic effect on osteoblasts and the liver, which reduces the production of the active form of vitamin D3. Drinking more than three units of alcohol per day is a risk factor for fractures [28]. On the other hand, some studies have shown that drinking small amounts of alcohol, combined with a healthy lifestyle, can have a positive effect on bone density [29,30].

**Smoking**. Smoking is a widely recognized risk factor for osteoporosis [31]. It causes a decrease in bone strength, more often in men than in women. This negative effect depends on how many cigarettes a person has smoked during their lifetime.

**Coffee**. Some recent studies have not shown that moderate coffee drinking increases the risk of osteoporosis and fractures in healthy adults (see [32] and the references therein). Caffeine, similar to theine, flushes out calcium and increases its excretion in the urine. However, the more calcium is washed out, the greater its absorption from food. It is therefore emphasized to provide adequate amounts of calcium with food. A recent review showed that there was a non-linear relationship between the level of coffee consumption and the incidence of hip fractures, and the lowest relative risk of hip fracture was found in those who consumed two to three cups of coffee per day [33].

**Glucocorticoids**. Taken chronically for more than 3 months, in a dose equivalent to 5 mg of prednisone daily, they may reduce bone formation, especially in the trabecular bone (vertebrae), increase urinary calcium excretion, and cause some hormonal disorders. Glucocorticoid-induced osteoporosis (GIO) is a common, iatrogenic, secondary osteoporosis that may be associated with a very high risk of fractures [2].

**Physical activity**. Various physical activities are effective in preventing and treating osteoporosis. The mechanical load resulting from physical activity increases muscle mass, creates stress on the skeleton, and increases osteoblast activity [22]. With age, skeletal muscle mass and function decrease, which, in the presence of osteoporosis, may increase the risk of falls and fractures. Strength training is recommended, which can be used without age restrictions.

**Sun exposure**. In our latitude (in Poland), from May to September, the synthesis of vitamin D3 in the skin can be effective from 10 a.m. to 3 p.m., in sunny weather, when at least the forearms and lower legs are exposed to the sun for at least 15 min without using sunscreen. Older people are at a high risk for vitamin D deficiency [34].

**Rheumatic diseases**—including rheumatoid arthritis, spondyloarthritis, and other connective tissue diseases. In inflammatory rheumatic diseases, there is a significant increase in the risk of osteoporosis and fractures [35]. Pro-inflammatory cytokines cause periarticular osteoporosis and activate osteoclastogenesis. The use of glucocorticoids may increase bone resorption. Additionally, joint damage, atrophy, and muscle weakness increase the risk of falls.

**Diabetes**. It is known that diabetic patients have increased risk of fractures. For patients with type 1 diabetes, BMD measurement should be performed 5 years after the diagnosis of the disease and repeated every 2–5 years. For patients with type 2 diabetes, the risk of fractures may not correspond to the BMD values [2]. Complications of diabetes such as myopathy, neuropathy, visual impairment, and obesity may increase the risk of falls.

**Neoplasma**—current or past. Cancer can have negative influence on bone health in many ways. Some cancer cells have an affinity for bone tissue, which results in bone metastases, leading to possible fractures. In addition, many cancer treatments can have detrimental effects on bone health. In particular, hormone deprivation therapies for breast cancer and prostate cancer adversely affect bone turnover, resulting in decreases in BMD and bone quality, which can lead to fractures [36].

**Hyperthyroidism**. Hyperthyroidism (also hyperparathyroidism), especially if left untreated for a long time, may contribute to bone atrophy, weakening of bone strength, and fractures [37]. Treatment of carcinoma of glandoma thyroidea with high doses of L-thyroxine in postmenopausal women increases fracture and osteoporosis risk.

**Hypogonadism or premature menopause** (<45 years). Postmenopausal women with estrogen deficiency have increased rate of remodeling of bones [27], particularly in the first 10 years after the last menstrual period. Low calcium absorption is observed, and the release of calcium from the bones is augmented. Secretion of parathyroid hormone reduces, and vitamin D3 metabolism decreases. Hypogonadism, low testosterone levels in elderly men are risk factors of bone resorption and fractures [38]. Ablative treatment of prostate cancer causes higher risk, independently of age.

**Gastrointestinal diseases**. Patients with gastrointestinal diseases are at high risk for osteoporosis and fractures [39]. Resection of the stomach and intestines and treatment with PPIs (proton pump inhibitors) may decrease the absorption of calcium and other nutrients. Using treatment with TNF-alpha inhibitors can reduce inflammation and bone resorption.

**Chronic kidney disease**. Due to biochemical abnormalities in the homeostasis of calcium and phosphorus, different types of osteodystrophy are distinguished. In the beginning, there is gradual retention of phosphorus and impairment of vitamin 1.25 OH D3. This disease significantly increases the risk of fractures [40].

**Strumectomy**. It is known that thyroidectomy significantly increases the long-term risk of osteoporosis. Younger patients, women, patients with comorbidities, and patients receiving chronic thyroxin treatment should be monitored for changes in postoperative bone density [41].

**Secondary osteoporosis**. The following diseases are included: type I (insulin dependent) diabetes, osteogenesis imperfecta in adults, untreated long-standing hyperthyroidism, hypogonadism or premature menopause (<45 years), chronic malnutrition or malabsorption, and chronic liver disease [3].

**Meat**. A meat diet is rich in saturated fats. The PRAR-gamma nuclear receptor can be activated by ligands, mainly products of oxidation of polyunsaturated fatty acids. This is responsible for an increase in adipocytogenesis. Lipoxygenase products bind to low-density lipoproteins (LDL) and increase osteoblast apoptosis. It has been shown that more bone fractures occur in countries with a high intake of animal protein. Studies have confirmed the bone-protective effect of a diet based on fruits and vegetables [42].

**Saltibg**. It is emphasized that reducing the amount of salt in the diet can help normalize blood pressure and reduce urinary calcium excretion. However, it was also observed that a low-salt diet can lead to a negative calcium and magnesium balance which could result in osteoporosis [43].

**Family history of hip fractures**. It has been suggested that the degree of bone remodeling and geometry may depend on genetic factors [44]. The occurrence of hip fractures in the immediate family is included among the clinical factors in the FRAX form [3].

**Dual-energy X-ray absorptiometry (DXA) neck T-score and dual-energy X-ray absorptiometry (DXA) spine T-score**.

To date, there is no known method that could measure the mechanical strength of bones. Currently, the diagnosis of osteoporosis is based on the DXA densitometry test, which measures bone density. A T-score (either neck or spine) of −2.5 SDs and below in postmenopausal women and men over 50 is classified as osteoporosis.

**Phosphates**—phosphate level in blood in mmol/L. Of the phosphorus in the human body, 85% is found in bones and teeth. It is available in many foods and drinks. Its bioavailability is in the range of 60–70%. Hyperphosphatemia reduces the absorption of calcium in the gastrointestinal tract, inhibits the synthesis of vitamin 1.25 (OH)2 D3 and independently increases the secretion of PTH. Therefore, the normal ratio of calcium to phosphorus is considered to be 1.5:1 or 1:1. Products that contain phosphorus include: meat, eggs, fish, lentils, beans, wheat bran, nuts, seeds, and carbonated drinks [42].

**Vitamin D3**—vitamin D3 level in blood in ng/mL. The special role of vitamin D3 is emphasized, which enables the absorption of calcium from the gastrointestinal tract, and also reduces the secretion of parathyroid hormone. Therefore, it helps in the regulation of calcium and phosphate metabolism. The diet covers only about 20% of the demand; thus, skin synthesis is important. When sufficient sun exposure is not possible, especially in people over 65, appropriate supplementation of this vitamin should be used throughout the year [42].

**Calcium**—calcium level in blood in mmol/L. The main inorganic component of bones is calcium. Approximately 99% of this macroelement in the body is accumulated here. Adequate supply during childhood and adolescence determines high peak bone mass. The body absorbs from 10 to 40% of calcium from the diet, and absorption from the intestines decreases with age. In adults, the demand for calcium is about 1000 mg/day. In the Polish population, the supply of this element is 50–60%. Supplementation with calcium preparations alone does not reduce bone fractures, but the risk of heart attack and kidney stones may increase [45].

### 3.3. Data Preparation

First, the features were divided into features that were to be input values to the model and features that were to be output data. It was necessary to decide how to take into account the results of subsequent medical tests. One test set consists of five parameters: the results of the densitometric examination of the L1–L4 vertebrae and the proximal femoral epiphysis, as well as laboratory tests of the level of 25 OH vitamin D3, calcium, and phosphate in the blood. For some people it was performed more than once. Initially, only the results of the first test were considered in this study.

A dataset used for the study included a group of 222 patients. Some of them had incomplete first set of tests. Therefore, 177 patients were chosen who had complete the first test set.

We used the following set of 27 features as input variables for ANNs:

1. Sex (M–male, F—female),
2. Age (in years),
3. BMI (real value),
4. Last menstruation (age in years),
5. Alcohol (Y—yes, N—no, S—in small amounts),
6. Smoking (Y—yes, N—no, S—at most 1 cigarette daily),
7. Coffee (number of cups daily),
8. Treatment with glucocorticoids (Y—yes, N—no),
9. Physical activity (≥ 30 min daily) (Y—yes, N—no),
10. Sun exposure (≥ 15 min daily) (Y—yes, N—no),
11. Rheumatic diseases (Y—yes, N—no),
12. Diabetes (Y—yes, N—no),
13. Neoplasma (Y—yes, N—no),
14. Hyperthyroidism (Y—yes, N—no),
15. Hypogonadism or premature menopause (Y—yes, N—no),
16. Gastrointestinal diseases (Y—yes, N—no),
17. Chronic kidney disease (Y—yes, N—no),
18. Strumectomy (Y—yes, N—no),
19. Secondary osteoporosis (Y—yes, N—no),
20. Meat in the diet (Y—yes, N—no, S—in small amounts),
21. Salting (Y—yes, N—no, S—in small amounts),
22. Family history of hip fractures (Y—yes, N—no),
23. DXA neck T-score (real value),
24. DXA spine T-score (real value),
25. Phosphates serum level (real value),
26. Vitamin D3 serum level (real value),
27. Calcium serum level (real value).

The set defined in this way includes features that take numerical values, are specified with a letter, or are empty. The last case concerns a feature that is not possible to determine for a given patient (e.g., last menstruation in men) and its absence must be described in some way. The following assumptions were made. The numerical data remained unchanged. Data described with values *Y*—yes, *N*—no, *S*—in small amounts, were converted to numerical values as follows: Y=1, N=−1 and S=0. If only two values were allowed: *Y*—yes, *N*—no, we assumed Y=1, N=−1. For features that are not possible to determine, the empty spaces have been filled with zeros. We understand such a feature value as the “not applicable” case. The above operations on the data allowed us to create a set consisting of complete numerical vectors. It was assumed that only one parameter would correspond to the output value. The value of this parameter was −1 if no fractures occurred and 1 if fractures occurred.

For each feature, the range of values that this feature takes was defined.

In addition to the above 27 features, our dataset contained also the following data for each patient:

- Number of low-energy fractures before treatment (FrBT),
- Number of low-energy fractures during treatment (FrDT).

This last information was used to train the neural network to correctly predict fractures in patients. The first analysis of the data was performed on the number of fractures that occurred before treatment. It was assumed that if the number of fractures was 0 (that is, FrBT had a value of 0), then the patient data was labeled −1 (the so-called ground truth label). If fractures before treatment occurred (that is, FrBT ≥1), the data was labeled 1. A correctly trained network should give the output −1 in case of fractures and 1 in case of no fractures. On this basis, a training set was created and a neural network was built.

An example of the feature vector for one patient is of the form:

(1) [F,70,22.58,50,N,N,0,N,Y,N,N,N,N,N,N,Y,N,Y,N,Y,N,N,−3.3,−3.3,1.13,73.2,2.5].

It has 27 components corresponding to subsequent items of the numbered list above. After converting the feature vector into a vector consisting of numerical data, we get the following input vector:

(2) [1,70,22.58,50,−1,−1,0,−1,1,−1,−1,−1,−1,−1,−1,1,−1,1,−1,1,−1,−1,−3.3,−3.3,1.13,73.2,2.5].

It is necessary to indicate the interval of acceptable values for each feature. Analyzing the data, we obtain 27 intervals defining the ranges of acceptable values for the relevant features: [0,1], [18,97], [16.02,45.01], [0,61], [−1,1], [−1,1], [0,4], [−1,1], [−1,1], [−1,1], [−1,1], [−1,1], [−1,1], [−1,1], [−1,1], [−1,1], [−1,1], [−1,1], [−1,1], [−1,1], [−1,1], [−1,1], [−4.1,1.6], [−4.8,3], [0.55,4.1], [5,110.5], [1.22,10.1].

**Remark** **1.** *Defining ranges for each feature is especially important. It should be remembered that these should be the maximum ranges from which the given features can come, and not the ranges determined by the minimum and maximum values from the observation data. One should analyze each feature and determine the entire domain for that feature. An example of this is the “age” feature. Regardless of the data present in the studied dataset, if the set of adults is considered, the range from which data are acceptable can be defined as 18 to 97 years.*

The next element is to define the output vector. In our case, the output is a value from the set {−1,1}. So, the output from the network should be −1 or 1.

### 3.4. Learning Neural Network

**Figure 1:**
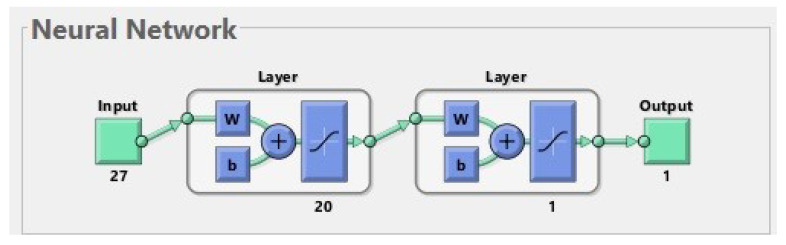
The best neural network for 177 patients. The input is composed of 27 features. The first layer (hidden layer) has 20 neurons; for simplicity, only one of them is shown in the diagram. The second layer (output layer) has 1 neuron. The output has 1 parameter (prediction of fractures before treatment).

An ANN is a simplified model of the structure of the brain. In our study, we used a multilayer feed-forward neural network, whose structure is shown in Figure 1. It is composed of artificial neurons which are organized in layers. Matlab software was used to create the ANN and carry out the learning procedure. The effectiveness of the network was checked on a testing set. During the testing procedure, it was verified whether the output generated by the ANN for a patient from the test set was consistent with the ground truth label containing the information about real fractures in this patient.

There are a number of methods that are used to implement the learning procedure. In this study, the method of back propagation was used.

Test data are necessary to check whether the previously trained network correctly generates a response for other data than those used in the learning process. The training set and the test set are created by appropriate division of the complete dataset. In this study, it is a set of 177 items that is split 80% to 20%, training data to test data. This division should be made many times in the above proportion and the training and testing procedure should be repeated many times on a possibly large group of different divisions of the dataset. In other words, we should train many different networks with many different training sets and validate them on different test sets. Each of these attempts should be repeated several times. The method can be considered effective if, for a given network structure, the average error on different test sets is small (<10%).

**Figure 2:**
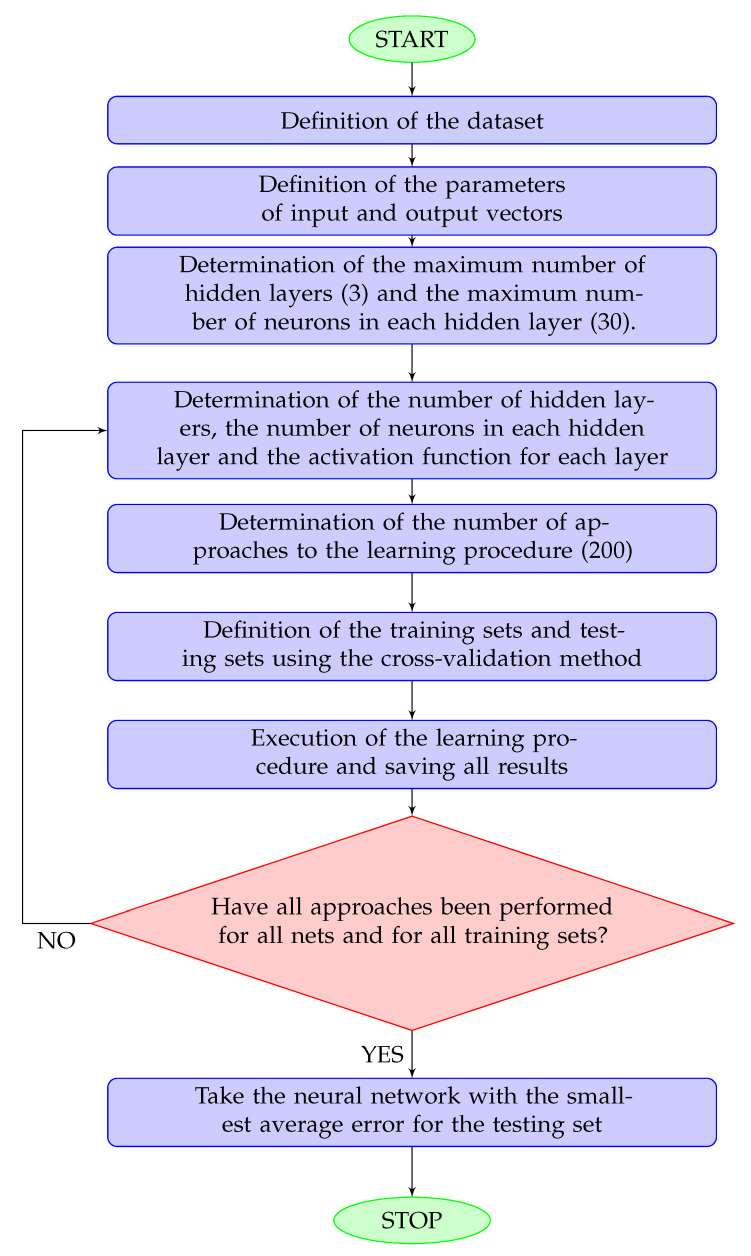
Flowchart for the network training procedure. A hidden layer is any layer other than the output layer. The maximum numbers of hidden layers and neurons was determined according to the size of the dataset.

The training procedure is explained in details in Figure 2.

As a result of the computational procedure, it was noticed that even single-layer networks learned the training data very well (with an error close to zero). It was much worse with data generalization: the error on test data exceeded 30%. Subsequent changes in the construction of the network did not lead to a significant improvement. Therefore, the elimination of individual features was used to check whether removing any of them will significantly affect the obtained results; however, this did not significantly affect the network error. In addition, an analysis of the relationships between individual features was performed, taking feature pairs and feature triples. This procedure did not lead to the detection of significant dependencies. Because the elimination of features and the analysis of selected features did not bring any significant changes in the obtained results, the best one-way two-layer network was adopted as the optimal solution.

### 3.5. The Structure of the Best Neural Network

The best network has 27 inputs. It consists of two layers with a sigmoidal activation function for each of them. The first layer consists of 20 neurons, and the last (second) layer consists of one neuron. On each layer, we use a hyperbolic tangent sigmoid transfer function $tansig\left(x\right)=\frac{2}{1+\exp\left(-2x\right)}-1$.

## 4. Results

Table 2, Table 3 and Table 4 describe the basic characteristics of patients of the osteoporosis treatment clinic whose data are taken into account. For each risk factor and for each category (e.g., Sex—Female and Sex—Male), we have computed the percentage of patients who had incident osteoporotic fractures, and the percentage of patients without such fractures. This provides information on the possible influence of individual risk factors on the likelihood of fractures. In Table 2, we have gathered information about sex, age, BMD, BMI, and laboratory tests. Table 3 contains the risk factors related to past or current diseases, and Table 4 contains factors related to patient’s lifestyle (these are the so-called modifiable risk factors).

The following notes apply to Table 2, Table 3 and Table 4.

1. N—number of patients in a given group.
2. The % sign in the table header means that the percentages in this column add up to 100% within a given risk factor.
3. The sign %* in the table header means that the percentages of patients with and without fractures in each row add up to 100%.

In Table 2, we have the following ranges of testing standards in the laboratory:

- serum phosphorus: 0.81–1.45 mmol/L,
- serum calcium level: 2.2–2.65 mmol/L,
- vitamin 25(OH)D3 level: 30–100 ng/mL.

In Table 4, ’smoking little’ means at most one cigarette daily.

### 4.1. The Results Calculations for the Set of 177 Patients

Calculations were made in Matlab. The following assumptions were made:

- The multilayer neural network described in Section 3.5 was used,
- The back-propagation method was used to realize the learning procedure,
- The mean squared error (MSE) was used to calculate the learning error.

MSE measures the average of the squares of the errors. It is a measure of quality of an estimator. MSE is defined as

(3) $$MSE=\frac{1}{n}\sum_{i=1}^{n}{\left(y_{i}-t_{i}\right)}^{2},$$

where *n* is the number of elements in a dataset, $\left\{y_{1},\cdots ,y_{n}\right\}$ is the set of predicted values, and $\left\{t_{1},\cdots ,t_{n}\right\}$ is the set of observed values.

The following results were obtained:

- The error for the training data: 0.000004,
- The error for the testing data: 0.344194.

### 4.2. Conclusions from the Calculations

After performing the calculations, the following conclusions can be drawn:

- The network approximates the training set correctly (with an error close to 0),
- The error for the testing data is too big; this means that the trained network generates incorrect output values for inputs other than from the training set,
- Additional data analysis is required to achieve better results for the test data.

Continuing the training and testing of the network without further analysis of the data may not bring new, better results. It can be expected that adding new patients to the existing dataset could favorably affect the result. For the data we currently have, it is necessary to use additional methods that extend and complete the results received from the neural network.

### 4.3. Further Analysis of the Dataset

Our initial attempts with the neural network, although successful during training, fell short when applied to test data. Because of these inconsistencies, we are now exploring other methods to better analyze our dataset. Essentially, we are testing different scientific techniques to discover the most efficient way to understand our data.

#### 4.3.1. PCA—Principal Component Analysis

Principal component analysis (PCA) is a technique used to simplify complex, multidimensional data into a space with fewer dimensions, making it easier to interpret. Through PCA, we have taken 27-element vectors—imagine these as different characteristics or variables—and converted them into a 2D space, similar to condensing a multi-volume encyclopedia into a summary booklet.

Our visualization of this transformed data was depicted as a graph, with two categories—those with fractures and those without—represented by different colors. The distribution of data across the graph was scattered, indicating that a clear division between these two groups was not feasible. A visual representation of this data distribution will be provided later, using a more extensive dataset.

#### 4.3.2. t-SNE—t-Distributed Stochastic Neighbor Embedding

t-distributed stochastic neighbor embedding (t-SNE) is a technique that is mainly used to visualize complex, multidimensional data. However, unlike PCA, t-SNE has a limitation—it can only visualize a fixed set of data, and integrating new data into this set is not a straightforward process.

Due to this limitation, it is often suggested to use PCA first to decrease the complexity of the data to a manageable level (preferably less than 50 features) before applying t-SNE. When we applied t-SNE to our original dataset, the data was also converted into a 2D space. Yet, similar to the PCA method, our data visualization indicated a scattered distribution throughout the space, with no clear path to distinguish between distinct groups. We will provide a visual representation using the t-SNE method later for a better understanding. In simpler terms, it is similar to trying to segregate a mixed crowd into distinct groups; that is how our data distribution looks.

#### 4.3.3. Binary Classification Methods

Binary classification methods leverage supervised learning to address the task of dividing a given set of data into two separate classes, typically labeled as positive and negative. In the context of our research, the ’positive’ class refers to patients with fractures, whereas the ’negative’ class comprises patients without fractures. The concept of supervised learning aligns closely with the neural network training process we previously discussed.

We are going to deploy three specific methods:

- Logistic regression (LR),
- *K*-nearest neighbors,
- Support vector machines (SVM).

In layman’s terms, think of supervised learning as teaching a machine using examples, similar to teaching a child how to identify animals by showing them pictures. Binary classification is similar to sorting objects into two baskets - in our case, we are sorting patients into two groups: those with fractures (’positive’) and those without fractures (’negative’). The three methods we will use are different strategies to do this sorting task.

#### 4.3.4. Metrics for Evaluating the Results

We will employ certain metrics to assess the results we have obtained. To do this, we first need to compute something called a confusion matrix (see Table 5); think of it as a scorecard for our binary classification model. This matrix is similar to a table that helps us understand the performance of our classification by comparing predicted and actual categories, both labeled as ’positive’ and ’negative’. It is possible that data originally tagged as ’positive’ might get mislabeled as ’negative’, or vice versa—the confusion matrix helps us track these errors.

We will then use the following metrics, explained in the context of binary classification.

**Precision:** this metric answers the following question: among all the data points we labeled as ’positive’, how many actually were positive? The precision formula is TP/(TP + FP), where TP stands for true positives (the correctly labeled positives) and FP stands for false positives (the negatives mislabeled as positives).

**Recall or sensitivity:** recall is used to determine the effectiveness for identifying actual positives. It is the proportion of true positive instances were identified. The recall formula is TP/(TP + FN), where FN stands for false negatives (the missed positives).

**Specificity:** specificity is used to determine the effectiveness for the identification of actual negatives. It is the proportion of true negative instances that were identified. The specificity formula is TN/(TN + FP), where TN stands for true negatives (the correctly labeled negatives).

**F1 score:** this is a combined metric that considers both precision and recall. It is used when the aim is to balance the two and achieve a model that is good at both identifying actual positives and avoiding false alarms. The F1 score formula is

(4) 2∗(precision∗recall)/(precision+recall).

By using these measures, we can obtain a nuanced picture of how well our classification methods are performing, rather than just a single score.

#### 4.3.5. Logistic Regression

We applied logistic regression, a binary classification method, to the dataset under consideration. In layman’s terms, this method tries to calculate the likelihood that a data point belongs to the ’positive’ category, allowing us to sort observations into one of two classes. This is performed by fitting a logistic function.

The logistic function we used is written as:

(5) $$f\left(x\right)=\frac{1}{1+e^{-(a\cdot x+b)}},$$

where a·x represents the scalar product of 27-dimensional vectors *a* and *x*, and *b* is a real number. During the learning phase, we adjust the values of *a* and *b* to maximize the correct assignments of data points to their respective classes.

To select the best model, we applied a technique known as *k*-fold cross-validation. We divided the entire dataset into *k* subsets, used one as the test set and the rest as the training set, and then performed the learning procedure. This process was repeated *k* times so that each subset served as the test set once. In our case, k=10.

Next, we tried to enhance our results using a process called recursive feature elimination. The aim was to find a smaller, more significant set of features that might improve the model, especially for test samples. This process involved eliminating one feature at a time then repeating the learning and results evaluation on the reduced feature set. This helped us rank the features from most to least important.

Following this process, we obtained the following ranking for the case where one feature was omitted. Some features were ranked as ’1’, indicating they were essential for an optimal model. Others were assigned different ranks, indicating their levels of significance (the larger the rank, the less significant the feature).

1. Neck T-score. 1. Chronic kidney disease. 2. Phosphates. 3. BMI. 4. Age. 5. Diet (meat). 6. Vitamin D3. 7. Calcium. 8. Strumectomy. 9. Hyperthyroidism. 10. Smoking. 11. Rheumatic diseases. 12. Last menstruation. 13. Cancer. 14. Diabetes. 15. Coffee. 16. Gastrointestinal diseases. 17. Glucocorticoids. 18. Sex. 19. Secondary osteoporosis. 20. Hypogonadism or premature menopause. 21. Alcohol. 22. Physical activity. 23. Salting. 24. Sun exposure. 25. Family history of hip fractures. 26. Spine T-score.

It should be noted that most features have been rejected and the number of significant features is very small.

For a set limited to two features, we applied the *k*-fold cross-validation method for k=5. The average result of F1 test was equal to 0.628426978.

### 4.4. Results for an Extended Set of Vectors

We extended our research to include a broader dataset encompassing 207 patients. Those previously excluded patients who had a full set of tests conducted at any subsequent date were reincorporated. For these reincorporated patients, we considered the first comprehensive result of two densitometry tests and the first full result of three lab tests. Moreover, we modified our approach in this study by replacing the FrBT variable with the sum of two variables: FrBT and FrDT. Hence, our interest lays in identifying if a patient experienced a low-energy fracture before or during the treatment process. Upon repeating our earlier computations with this expanded dataset, we generated a new set of results. These will be discussed in the following section.

#### 4.4.1. Neural Networks

The calculated mean squared error (MSE) on the expanded test set of 41 items (20% of the total data) continues to remain high, at 33%.

#### 4.4.2. PCA—Principal Component Analysis

**Figure 3:**
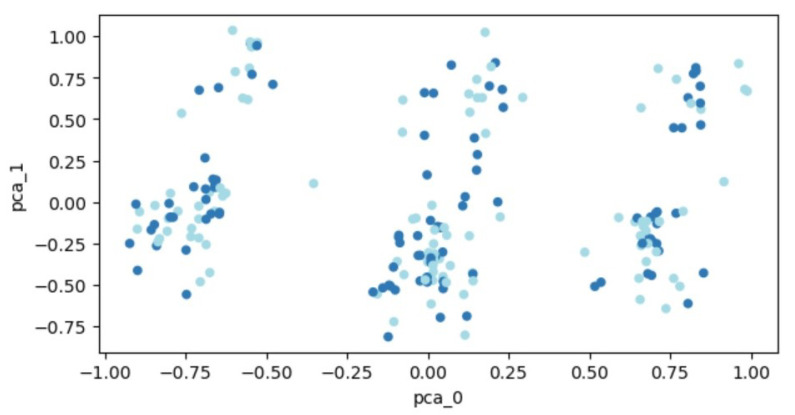
PCA outcomes for the expanded set of 207 patients. Each dot represents a patient’s feature vector transformed into a two-dimensional space. Varying colors are used to differentiate between patient groups: those who experienced fractures and those who did not.

The transformed data, following PCA, is visualized in Figure 3. Patient data are plotted as points within a two-dimensional space. Two distinct groups of points, representing individuals with and without a fracture, are depicted in different colors. However, the distribution of data points across the entire space makes it challenging to demarcate distinct regions, thereby hindering the separation of the two classes.

#### 4.4.3. t-SNE—t-Distributed Stochastic Neighbor Embedding

**Figure 4:**
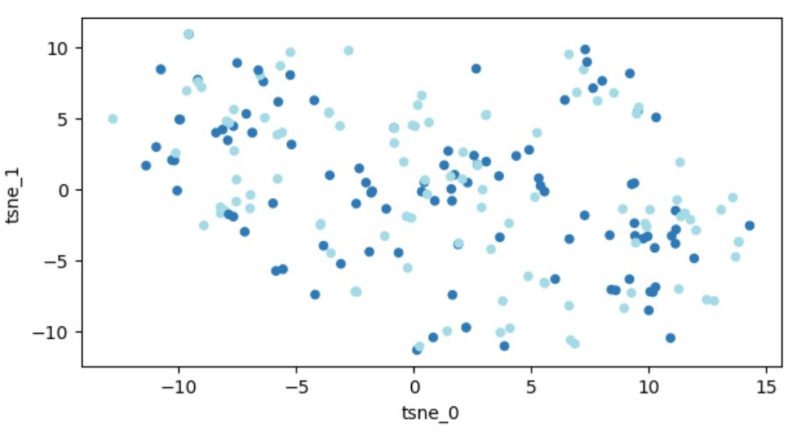
Outcomes of t-SNE application on the dataset of 207 patients. Every dot signifies a feature vector related to one patient expressed within a two-dimensional framework. Variations in colors are employed to distinguish different patient groups (those with fractures and those without).

A graphic illustration of our data is demonstrated in Figure 4. Similar to before, the data are distributed throughout the entire space, indicating that segregating the two classes remains an unattainable task.

#### 4.4.4. Logistic Regression

With the implementation of the recursive feature elimination process, we established a hierarchical ranking for the features. Again, features ranked as ’1’ were essential for an optimal model:

1. Age. 1. Chronic kidney disease. 1. Neck T-score. 1. Phosphates. 2. Diet (meat). 3. BMI. 4. Smoking. 5. Strumectomy. 6. Hyperthyroidism. 7. Calcium. 8. Vitamin D3. 9. Rheumatic diseases. 10. Spine T-score. 11. Secondary osteoporosis. 12. Hypogonadism or premature menopause. 13. Sex. 14. Last menstruation. 15. Family history of hip fractures. 16. Coffee. 17. Gastrointestinal diseases. 18. Glucocorticoids. 19. Sun exposure. 20. Diabetes. 21. Alcohol. 22. Physical activity. 23. Cancer. 24. Salting.

Limiting the set to the first four features and applying the *k*-fold cross-validation technique (k=5), the mean F1 test outcome was approximately 0.652854936.

The AUC (area under the ROC curve) of the LR model is 0.54, which suggests that the model has a 54% chance of correctly distinguishing between positive and negative classes. This is better than random guessing (which would have an AUC of 0.5), but not particularly strong.

The sensitivity (also known as the true positive rate or recall) of the LR model is 0.59. This means that the model correctly identifies 59% of the positive instances.

The specificity (also known as the true negative rate) of the LR model is 0.65. This means that the model correctly identifies 65% of the negative instances.

#### 4.4.5. *K*-Nearest Neighbors

The *K*-nearest-neighbors (*K*-NN) approach was another method applied. In this method, the input comprises the *K* nearest training examples within a dataset, with *K* being a positive integer. The output reflects class membership: an object’s classification is determined by a majority vote from its neighbors, being assigned to the most prevalent class among its K nearest neighbors.

Compared to the regression method, the *K*-NN method, yielded significantly lower results, considering the F1 test score. With different *K* values defining the number of neighbors, the best outcome was achieved for K=28, in which case the F1 test result was approximately 0.60606.

The AUC of the *K*-NN model is 0.54, the same as for the LR model.

The sensitivity of the *K*-NN model is 0.5, which means that the model correctly identifies 50% of the positive instances. 

The specificity of the *K*-NN model is 0.6, which means that the model correctly identifies 60% of the negative instances.

#### 4.4.6. Support Vector Machines

The final method applied was the support vector machine (SVM) method. An SVM is a supervised learning algorithm aimed at distinguishing two separate classes of points either by a hyperplane in the original space or in some transformed space, often of higher dimension. These transformations can be accomplished using different kernel functions.

We utilized an SVM method with various kernel functions, with the best result obtained using the radial function. In this instance, the F1 test outcome was approximately 0.666666.

The AUC of the SVM model is 0.53, which is slightly better than random guessing but still not particularly strong.

The sensitivity of the SVM model is 0.32, which means that the model correctly identifies only 32% of the positive instances. This is the lowest sensitivity among the three models.

The specificity of the SVM model is 0.65, which means that the model correctly identifies 65% of the negative instances.

#### 4.4.7. Further Application of Neural Networks

**Figure 5:**
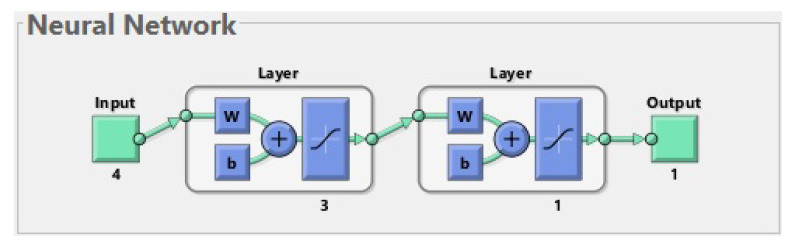
The best neural network for 207 patients. The input is composed of 4 features (age, chronic kidney disease, neck T-score, and phosphates). The first layer (hidden layer) has 3 neurons. The second layer (output layer) has 1 neuron. The output has 1 parameter (prediction of fractures before or during treatment).

In order to further reduce the error on the test set, the learning process of the neural network was repeated, taking into account only those four features that were assigned ones in the previous ranking. Various possible network structures were investigated. The best was a two-layer network, where there were three neurons in the hidden layer and one in the output layer (see Figure 5).

The value of the acceptable error on the training set was set at 0.1. The average MSE obtained on the test set was 0.2668.

## 5. Discussion

### Interpretation of the Above Results

Table 6 presents a comparison of results obtained for the first neural network (see Section 4.1) and the second neural network (see Section 4.4.7).

Concerning other classification methods used in this study, in Table 7, we show the F1 scores for them (for the set of 207 patients and four features).

The F1 score, which is the harmonic mean of precision and recall and gives a balanced measure of model performance, was calculated for each of the models. The LR model yielded an F1 score of 0.6528, indicating a balanced trade-off between precision and recall, and implying that it correctly identified 65.28% of positive instances while maintaining a low rate of false positives. The *K*-NN model produced an F1 score of 0.6060, which is slightly lower than that of LR. This result suggests that although the *K*-NN model was able to correctly classify a reasonable proportion of instances, it did not perform as well as the LR in terms of balancing false positives and false negatives. This could be due to the nature of the *K*-NN algorithm, which classifies an instance based on the majority class of its nearest neighbors and might not perform as well if the dataset has complex or non-linear relationships that cannot be captured by simple distance measures. On the other hand, the SVM model had the highest F1 score of 0.666, indicating that it was able to achieve the best balance between precision and recall among the three models. SVMs are known for their ability to handle high-dimensional data and to find optimal boundaries between classes, which might explain the higher performance in this case. Although the results suggest that the SVM model provided the best performance according to the F1 score, it is crucial to note that the choice of model should be made considering the specific context and requirements of the task. For example, if the cost of false positives is very high, one might prefer a model with a higher precision even at the cost of recall, and vice versa.

Finally, it is also important to consider other metrics such as the AUC-ROC, sensitivity, and specificity, which provide different perspectives on model performance. All three models have similar AUC values around 0.53–0.54, which suggests that they are not highly effective at distinguishing between positive and negative classes. The logistic regression and SVM models have the highest specificity of 0.65, whereas the logistic regression model has the highest sensitivity of 0.59. Therefore, if the goal is to maximize sensitivity (i.e., correctly identifying positive instances), the logistic regression model may be the best choice among these three. However, overall, these models may need further tuning or additional features to improve their performance.

The considered group of patients was small: in some subgroups, there were only several people with a given risk factor. Further cohort studies with more patients may be needed to identify additional risk factors and to develop a reliable fracture risk system.

One of the goals of our research was to identify fracture risk factors relevant to the Polish population. Similar studies have previously been conducted (see [46,47]) but none of them have used ANNs. Moreover, in [46], the influence of various risk factors on lumbar spine BMD was examined. In [47], the risk of osteoporotic fractures was assessed by the FRAX calculator. The results concerning significant risk factors presented in these two papers were also different than ours. According to [46], the significant risk factors of low lumbar spine BMD were age, BMI, year of menopause, and family history of osteoporosis. On the other hand, in [47], it was shown by using the LR method that the most significant risk factors of osteoporosis and fractures were: cigarette smoking, past gynecological operations, and corticosteroid therapies. Let us note, however, that the populations considered were also different: in [46], they were “women aged 30–79 years, representative of the general population”, whereas in [47], “the study was carried out with the female residents of 4 care facilities”, age 65 or older.

Our research is similar to [48]. In both papers, the authors focused on osteoporosis problems. Both refer to it in a different way, setting a different goal of the conducted research. The authors of both papers use various sets of methods. Despite some differences in these sets of methods, we can see that the most of them are the same. Based on the obtained results, it can be seen that they are comparable.

Finally, we want to highlight a possible direction of further research. We plan to use optimal control and differential equation tools to construct a family of functions defining a machine learning block, and a cost function defined on this family for determining a function which will assign patients to one of two groups: fractures or no fractures. Construction of the function will be performed by choosing an approximate optimal value to the function among neural networks (functions satisfying differential equations) learned from patterns found in the data from OTC. We hope that this function will allow us to predict fractures with an error less than 5%.

Because fractures are important risk factor of subsequent fractures (see the FRAX calculator and [2], Figure 2), further research is necessary where fractures are also considered as input data.

## 6. Conclusions

As a result of the first analysis of the set of 177 patients, the following most important risk factors were identified: neck T-score and chronic kidney disease. The second analysis of the set of 207 patients has shown that the following risk factors are most important: age, chronic kidney disease, neck T-score, and phosphates.

After using several methods, the following conclusions can be drawn.

- The applied statistical models as well as basic methods of neural networks are not satisfactory for predicting fractures based on a rather small set of patients. However, they may be successful in determining the hierarchy of risk factors. More advanced methods—such as deep learning—can possibly yield better results.
- Using the model for all features gives worse results than a model based on a dataset limited only to the most important features.
- The comparison of feature rankings obtained for different numbers of patients shows that the applied method of determining the hierarchy of risk factors is very sensitive to changes in the considered data (adding new patients significantly changes the result).

## Figures and Tables

**Table 1:** Diagnostic criteria for osteoporosis.

Norm	T-score ≥−1.0
Low bone mass (osteopenia)	−2.5< T-score <−1.0
Osteoporosis	T-score ≤ −2.5
Advanced osteoporosis	T-score ≤ −2.5
	and the presence of a low-energy fracture

**Table 2:** Characteristics of the cohort with respect to sex, BMI, BMD, and lab tests.

Risk Factor	Total		With Fracture		Without Fracture	
	**N**	**%**	**N**	%*	**N**	%*
**Sex**						
Female	206	92.8	101	49.0	105	51.0
Male	16	7.2	6	37.5	10	62.5
**Age**						
≤50 years	7	3.2	2	28.6	5	71.4
51–60 years	30	13.5	13	43.3	17	56.7
61–70 years	80	36.0	33	41.3	47	58.8
71–80 years	76	34.2	37	48.7	39	51.3
>80 years	29	13.1	22	75.9	7	24.1
**BMI**						
BMI < 18.5	6	2.7	4	66.7	2	33.3
18.5 ≤ BMI ≤ 24.9	124	55.9	55	44.4	69	55.6
BMI > 24.9	92	41.4	48	52.2	44	47.8
**DXA Neck T-score (T)**						
T ≥ −1	40	18.0	13	32.5	27	67.5
−2.5 < T < −1	131	59.0	61	46.6	70	53.4
T ≤ −2.5	51	23.0	33	64.7	18	35.3
**DXA Spine T-score (T)**						
T ≥ −1	51	23.0	20	39.2	31	60.8
−2.5 < T < −1	86	38.7	45	52.3	41	47.7
T ≤ −2.5	85	38.3	42	49.4	43	50.6
**Phosphates**						
Below the norm	3	1.4	1	33.3	2	66.7
Normal	204	95.8	97	47.5	107	52.5
Above the norm	6	2.8	5	83.3	1	16.7
**Vitamin D3** **(d)**						
d ≤ 20	24	10.9	11	45.8	13	54.2
20 < d ≤ 30	47	21.4	22	46.8	25	53.2
30 < d ≤ 50	101	45.9	49	48.5	52	51.5
50 < d ≤ 100	45	20.5	22	48.9	23	51.1
d > 100	3	1.4	2	66.7	1	33.3
**Calcium**						
Below the norm	2	0.9	1	50.0	1	50.0
Normal	113	96.8	102	47.9	111	52.1
Above the norm	5	2.3	2	40.0	3	60.0

**Table 3:** Characteristics of the cohort with respect to health conditions.

Risk Factor	Total		With Fracture		Without Fracture	
	**N**	**%**	**N**	%*	**N**	%*
**Age of last menstruation** **(m)**						
0 < m < 45	22	9.9	12	54.5	10	45.5
Still menstruating or m ≥ 45	184	82.9	89	48.4	95	51.6
**Hypogonadism or**						
**premature menopause**						
Yes	22	9.9	12	54.5	10	45.5
No	200	90.1	95	47.5	105	52.5
**Secondary osteoporosis**						
Yes	23	10.4	12	52.2	11	47.8
No	199	89.6	95	47.7	104	52.3
**Strumectomy**						
Yes	22	9.9	7	31.8	15	68.2
No	200	90.1	100	50.0	100	50.0
**Hyperthyroidism**						
Yes	13	5.9	6	46.2	7	53.8
No	209	94.1	101	48.3	108	51.7
**Rheumatic diseases**						
Yes	17	7.7	7	41.2	10	58.8
No	205	92.3	100	48.8	105	51.2
**Diabetes**						
Yes	22	9.9	10	45.5	12	54.5
No	200	90.1	97	48.5	103	51.5
**Neoplasma**						
Yes	31	14.0	13	41.9	18	58.1
No	191	86.0	94	49.2	97	50.8
**Gastrointestinal diseases**						
Yes	57	25.7	27	47.4	30	52.6
No	165	74.3	80	48.5	85	51.5
**Chronic kidney disease**						
Yes	5	2.3	5	100.0	0	0.0
No	217	97.7	102	47.0	115	53.0
**Glucocorticoids**						
Yes	20	9.0	8	40.0	12	60.0
No	202	91.0	99	49.0	103	51.0
**Family history of hip fractures**						
Yes	24	10.8	14	58.3	10	41.7
No	198	89.2	93	47.0	105	53.0

**Table 4:** Characteristics of the cohort with respect to lifestyle.

Risk Factor	Total		With Fracture		Without Fracture	
	**N**	**%**	**N**	%*	**N**	%*
**Alcohol**						
Yes	4	1.8	3	75.0	1	25.0
In small amounts	65	29.3	29	44.6	36	55.4
No	153	68.9	75	49.0	78	51.0
**Smoking**						
Smokers	23	10.4	15	65.2	8	34.8
Smoking little	3	1.4	1	33.3	2	66.7
Non-smokers	196	88.3	91	46.4	105	53.6
**Coffee (d cups daily)**						
d < 1	89	40.1	46	51.7	43	48.3
1 ≤ d < 2	77	34.7	36	46.8	41	53.2
2 ≤ d < 3	49	22.1	20	40.8	29	59.2
d ≥ 3	7	3.2	5	71.4	2	28.6
**Physical activity**						
Yes	157	70.7	74	47.1	83	52.9
No	65	29.3	33	50.8	32	49.2
**Sun exposure**						
Yes	72	32.4	33	45.8	39	54.2
No	150	67.6	74	49.3	76	50.7
**Meat in the diet**						
Yes	211	95.0	103	48.8	108	51.2
In small amounts	6	2.7	4	66.7	2	33.3
No	5	2.3	0	0.0	5	100.0
**Salting**						
Yes	55	24.8	30	54.5	25	45.5
In small amounts	79	35.6	32	40.5	47	59.5
No	88	39.6	45	51.1	43	48.9

**Table 5:** Confusion matrix.

		Actual Values	
		Positive	Negative
**Predicted Values**	Positive	True-Positive (TP)	False-Positive (FP)
	Negative	False-Negative (FN)	True-Negative (TN)

**Table 6:** Errors for the two neural networks.

MSE Results for the Neural Networks	1st Neural Network	2nd Neural Network
For the training data	0.000004	0.1
For the testing data	0.344194	0.2668

**Table 7:** F1 scores for different classification methods (for 207 patients and 4 features).

Methods	F1 Score
Logistic Regression	0.652854936
K-Nearest Neighbors	0.60606
SVM	0.666666

## Data Availability

Data sharing not applicable.

## Abbreviations

The following abbreviations are used in this manuscript:

AI	Artificial Intelligence
ANN	Artificial Neural Network
AUC	Area Under the ROC curve
BMI	Body Mass Index
BMD	Bone Mineral Density
DXA	Dual-Energy X-ray Absorptiometry
FN	False Negative
FP	False Positive
FRAX	Fracture Risk Assessment Tool
FrBT	Number of low-energy fractures before treatment
FrDT	Number of low-energy fractures during treatment
ICD	International Classification of Diseases
*K*-NN	*K*-Nearest Neighbors
LR	Logistic Regression
MSE	Mean Squared Error
OTC	Osteoporosis Treatment Clinic
PCA	Principal Component Analysis
PTH	Parathyroid Hormone
ROC	Receiver Operating Characteristic
t-SNE	t-distributed Stochastic Neighbor Embedding
SD	Standard Deviation
SVM	Support Vector Machines
TP	True-Positive
TN	True-Negative

## References

[1] Czerwinski E., Kanis J.A., Osieleniec J., Kumorek A., Milert A., Johansson H., McCloskey E.V., Gorkiewicz M. (2011). Evaluation of FRAX to characterise fracture risk in Poland. Osteoporos. Int..

[2] Głuszko P., Sewerynek E., Misiorowski W., Konstantynowicz J., Marcinowska-Suchowierska E., Blicharski T., Jabłoński M., Franek E., Kostka T., Jaworski M. (2023). Guidelines for the diagnosis and management of osteoporosis in Poland. 2022. Endokrynol. Pol..

[3] FRAX Fracture Risk Assessment Tool. https://frax.shef.ac.uk/FRAX/index.aspx.

[4] Miedany I.E. (2020). FRAX: Re-adjust or re-think. Arch. Osteoporos..

[5] Leslie W.D., Majumdar S.R., Lix L.M., Johansson H., Oden A., McCloskey E., Kanis J.A. (2012). High fracture probability with FRAX usually indicates densitometric osteoporosis: Implications for clinical practice. Osteoporos. Int..

[6] Gourlay M.L., Ritter V.S., Fine J.P., Overman R.A., Schousboe J.T., Cawthon P.M., Orwoll E.S., Nguyen T.V., Lane N.E., Cummings S.R. (2017). Comparison of fracture risk assessment tools in older men without prior hip or spine fracture: The MrOS study. Arch. Osteoporos..

[7] Cruz A.S., Lins H.C., Medeiros R.V.A., Filho J.M.F., da Silva S.G. (2018). Artificial intelligence on the identification of risk groups for osteoporosis, a general review. BioMed. Eng. OnLine.

[8] Discover the Advantages of FRAXplus. https://www.fraxplus.org/frax-plus.

[9] Ferizi U., Honig S., Chang G. (2019). Artificial intelligence, osteoporosis and fragility fractures. Curr. Opin. Rheumatol..

[10] Grace S.J., Kumar D.S., Gautam R., Sachin M.K. (2019). Osteoporosis detection using deep learning. Int. J. Mod. Trends Sci. Technol..

[11] Iliou T., Anagnostopoulos C.N., Anastasssopoulos G. (2014). Osteoporosis detection using machine learning techniques and feature selection. Int. J. Artif. Intell. Tools.

[12] Mantzaris D.H., Anastassopoulos G.C., Lymberopoulos D.K. Medical disease prediction using artificial neural networks. Proceedings of the 8th IEEE International Conference on BioInformatics and BioEngineering.

[13] Mantzaris D., Anastassopoulos G., Ilidis L., Konstantinos K., Papadopoulos H., Papadopoulos H., Andreou A.S., Bramer M. (2010). A soft computing approach for osteoporosis risk factor estimation. Artificial Intelligence Applications and Innovations 2010.

[14] Moudani W., Shahin A., Chakik F., Rajab D. (2011). Intelligent predictive osteoporosis system. Int. J. Comput. Appl..

[15] Sathawane K.S., Tuteja R.R. (2015). Data mining in clinical records to foretell the risk of osteoporosis. Int. J. Res. Advent Technol..

[16] Devikanniga D., Raj R.J.S. (2018). Classification of osteoporosis by artificial neural network based on monarch butterfly optimisation algorithm. Healthc. Technol. Lett..

[17] Reshmalakshmi C., Sasikumar M. Fuzzy inference system for osteoporosis detection. Proceedings of the IEEE 2016 Global Humanitarian Technology Conference.

[18] Hong C.M., Lin C.T., Huang C.Y., Lin Y.M. (2008). An intelligent fuzzy-neural diagnostic system for osteoporosis risk assessment. World. Acad. Sci. Eng. Technol..

[19] Shubangi D.C., Shilpa K. (2017). A survey on detection and diagnosis of osteoporosis. Int. J. Eng. Sci. Invention.

[20] Ji L., Zhang W., Zhong X., Zhao T., Sun X., Zhu S., Tong Y., Luo J., Xu Y., Yang D. (2022). Osteoporosis, fracture and survival: Application of machine learning in breast cancer prediction models. Front. Oncol..

[21] Bui H.M., Ha M.H., Pham H.G., Dao T.P., Nguyen T.T., Nguyen M.L., Vuong N.T., Hoang X.H.T., Do L.T., Dao T.X. (2022). Predicting the risk of osteoporosis in older Vietnamese women using machine learning approaches. Sci. Rep..

[22] Fasihi L., Tartibian B., Eslami R., Fasihi H. (2022). Artificial intelligence used to diagnose osteoporosis from risk factors in clinical data and proposing sports protocols. Sci. Rep..

[23] Kong S.H., Ahn D., Kim B.R., Srinivasan K., Ram S., Kim H., Hong A.R., Kim J.H., Cho N.H., Shin C.S. (2020). A novel fracture prediction model using machine learning in a community-based cohort. JBMR Plus.

[24] Suh B., Yu H., Kim H., Lee S., Kong S., Kim J.W., Choi J. (2023). Interpretable deep-learning approaches for osteoporosis risk screening and individualized feature analysis using large population-based data: Model development and performance evaluation. J. Med. Internet Res..

[25] Rinonapoli G., Ruggiero C., Meccariello L., Bisaccia M., Ceccarini P., Caraffa A. (2021). Osteoporosis in men: A review of underestimated bone condition. Int. J. Mol. Sci..

[26] Johnston C.B., Dagar M. (2020). Osteoporosis in older adults. Med. Clin. N. Am..

[27] Bagur A.C., Mautalen C.A. (1992). Risk for developing osteoporosis in untreated premature menopause. Calcif. Tissue. Int..

[28] Kanis J.A., Johansson H., Johnell O., Oden A., De Laet C., Eisman J.A., Pols H., Tenenhouse A. (2005). Alcohol intake as a risk factor for fracture. Osteoporos. Int..

[29] Kanis J.A., Johnell O., Oden A., Johansson H., De Laet C., Eisman J.A., Fujiwara S., Kroger H., McCloskey E.V., Mellstrom D. (2005). The effect of moderate alcohol consumption on bone mineral density: A study of female twins. Ann. Rheum. Dis..

[30] Godos J., Giampieri F., Chisari E., Micek A., Paladino N., Forbes-Hernández T.Y., Quiles J.L., Battino M., La Vignera S., Musumeci G. (2022). Alcohol consumption, bone mineral density, and risk of osteoporotic fractures: A dose–response meta-analysis. Int. J. Environ. Res. Public Health.

[31] Kanis J.A., Johnell O., Odén A., Johansson H., De Laet C., Eisman J.A., Fujiwara S., Kroger H., McCloskey E.V., Mellstrom D. (2005). Smoking and fracture risk: A meta-analysis. Osteoporos. Int..

[32] Berman N.K., Honig S., Cronstein B.N., Pillinger M.H. (2022). The effects of caffeine on bone mineral density and fracture risk. Osteoporos. Int..

[33] Zeng X., Su Y., Tan A., Zou L., Zha W., Yi S., Lv Y., Kwok T. (2022). The association of coffee consumption with the risk of osteoporosis and fractures: A systematic review and meta-analysis. Osteoporos. Int..

[34] Sözen T., Özışık L., Başaran N.Ç. (2017). An overview and management of osteoporosis. Eur. J. Rheumatol..

[35] Lee J.H., Suh Y.S., Koh J.H., Jung S.M., Lee J.J., Kwok S.K. (2014). The risk of osteoporotic fractures according to the FRAX model in Korean patients with rheumatoid arthritis. J. Korean Med. Sci..

[36] Ye C., Leslie W.D. (2023). Fracture risk and assessment in adults with cancer. Osteoporos. Int..

[37] Delitala A.P., Scuteri A., Doria C. (2020). Thyroid hormone diseases and osteoporosis. J. Clin. Med..

[38] Golds G., Houdek D., Arnason T. (2017). Male hypogonadism and osteoporosis: The effects, clinical consequences, and treatment of testosterone deficiency in bone health. Int. J. Endocrinol..

[39] Oh H.J., Ryu K.H., Park B.J., Yoon B.H. (2018). Osteoporosis and osteoporotic fractures in gastrointestinal disease. J. Bone Metab..

[40] Lima G.A.C., Paranhos-Neto F.P., Pereira G.R.M., Gomes C.P., Farias M.L.F. (2014). Osteoporosis management in patient with renal function impairment. Arq. Bras. Endocrinol. Metab..

[41] Hung C.L., Yeh C.C., Sung P.S., Hung C.J., Muo C.H., Sung F.C. (2018). Is partial or total thyroidectomy associated with risk of long-term osteoporosis: A nationwide population-based study. World J. Surg..

[42] Tucker K.L. (2009). Osteoporosis prevention and nutrition. Curr. Osteoporos. Rep..

[43] DiNicolantonio J.J., Mehta V., Zaman S.B., O’Keefe J.H. (2018). Not salt but sugar as aetiological in osteoporosis: A review. Mo. Med..

[44] Trajanoska K., Rivadeneira F. (2020). Genomic medicine: Lessons learned from monogenic and complex bone disorders. Front. Endocrinol..

[45] Kanis J.A., Cooper C., Rizzoli R., Reginster J.Y. (2019). European guidance for the diagnosis and management of osteoporosis in postmenopausal women. Osteoporos. Int..

[46] Filip R.S., Zagórski J. (2005). Osteoporosis risk factors in rural and urban women from the Lublin Region of Poland. Ann. Agric. Environ. Med..

[47] Nawrat-Szołtysik A., Miodońska Z., Zarzeczny R., Zając-Gawlak I., Opara J., Grzesińska A. (2020). Osteoporosis in Polish older women: Risk factors and osteoporotic fractures: A cross-sectional study. Int. J. Environ. Res. Public Health.

[48] Lin Y.T., Chu C.Y., Hung K.S., Lu C.H., Bednarczyk E.M., Chen H.Y. (2022). Can machine learning predict pharmacotherapy outcomes? An application study in osteoporosis. Comput. Methods Programs Biomed..

